# A fluorescence-based assay suitable for quantitative analysis of deadenylase enzyme activity

**DOI:** 10.1093/nar/gkt972

**Published:** 2013-10-28

**Authors:** Maryati Maryati, Ishwinder Kaur, Gopal P. Jadhav, Loyin Olotu-Umoren, Blessing Oveh, Lubna Hashmi, Peter M. Fischer, G. Sebastiaan Winkler

**Affiliations:** School of Pharmacy and Centre for Biomolecular Sciences, University of Nottingham, University Park, Nottingham NG7 2RD, UK

## Abstract

In eukaryotic cells, the shortening and removal of the poly(A) tail of cytoplasmic mRNA by deadenylase enzymes is a critical step in post-transcriptional gene regulation. The ribonuclease activity of deadenylase enzymes is attributed to either a DEDD (Asp-Glu-Asp-Asp) or an endonuclease–exonuclease–phosphatase domain. Both domains require the presence of two Mg^2+^ ions in the active site. To facilitate the biochemical analysis of deadenylase enzymes, we have developed a fluorescence-based deadenylase assay. The assay is based on end-point measurement, suitable for quantitative analysis and can be adapted for 96- and 384-well microplate formats. We demonstrate the utility of the assay by screening a chemical compound library, resulting in the identification of non-nucleoside inhibitors of the Caf1/CNOT7 enzyme, a catalytic subunit of the Ccr4–Not deadenylase complex. These compounds may be useful tools for the biochemical analysis of the Caf1/CNOT7 deadenylase subunit of the Ccr4–Not complex and indicate the feasibility of developing selective inhibitors of deadenylase enzymes using the fluorescence-based assay.

## INTRODUCTION

Accurate control of gene expression depends on the precise regulation of mRNA levels by both transcriptional and post-transcriptional mechanisms. A key step in the post-transcriptional regulation of mRNA levels involves the shortening of the poly(A) tail of cytoplasmic messenger RNA (mRNA) by deadenylase enzymes (1–5). These enzymes play an important role in mRNA turnover. In addition, deadenylation may also impact on translation as the relation between poly(A) tail length and translational efficiency is well established (6,7).

Around 10 deadenylases are encoded by the human genome (2). The catalytic activity of deadenylases is provided by either an endonuclease–exonuclease–phosphatase (EEP) domain, or a DEDD (Asp-Glu-Asp-Asp) fold. In both cases, deadenylation is dependent on the presence of two Mg^2+^ ions in the active site (2). Examples of EEP-type deadenylases include the circadian deadenylase Nocturnin/CCRN4L and the mitochondrial deadenylase PDE12 (5,8,9). In contrast, PARN, a homodimeric deadenylase that also contains a cap-binding domain, and Pan2, which forms a heterodimeric complex with Pan3, contain a DEDD domain (4,5,10–15). The composition of the Ccr4–Not complex, a major deadenylase important for cytoplasmic mRNA degradation (16–19), is unusually intricate as compared with other deadenylases (4,20,21). In addition to at least six non-catalytic subunits, the complex contains two distinct subunits with deadenylase activity: a Caf1 subunit containing a DEDD domain, and a Ccr4 component characterized by an EEP fold (4,22,23). Both enzymatic subunits are tethered to the non-catalytic components via the large subunit CNOT1. The centrally located MIF4G domain of CNOT1 contains multiple helical repeats that interact with the Caf1 catalytic subunit (24,25). In turn, a helix/loop region of Caf1 binds via hydrophobic interactions with the leucine-rich repeat domain of the Ccr4 deadenylase subunit (24). In vertebrate cells, the complexity of the Ccr4–Not deadenylase is further increased by the occurrence of two highly similar Caf1 paralogues (encoded by either *CNOT7* or *CNOT8*) (26,27). Similarly, the *CNOT6* and *CNOT6L* genes encode two Ccr4 paralogues associated with the Ccr4–Not complex in vertebrates (28). It is currently unclear to what extent the catalytic components of the Ccr4–Not complex have redundant or unique roles in mRNA deadenylation (29–31).

To obtain further insight into the cellular and physiological roles of deadenylase enzymes, novel tools such as potent, selective and cell-permeable inhibitors of deadenylase enzymes are desirable. Such molecules would be valuable as chemical probes and complement the use of RNAi-based tools, as they would inhibit the enzymatic activity rather than interfere with potential structural roles of deadenylase enzymes. Towards this goal, we first developed a new fluorescence-based deadenylase assay, because the various assays currently available for the biochemical analysis of deadenylase enzymes are time consuming, and less suitable for quantitative analysis and screening. For example, widely used gel-based assays based on (oligonucleotide) substrates labelled with fluorescent or radioactive moieties are difficult to quantify and are laborious. In contrast, quantitative assays based on methylene blue colourimetry are insensitive and require high protein and substrate concentrations (32,33). Finally, recently developed quantitative assays based on size-exclusion chromatography also have limited sensitivity, require relatively large reaction volumes and are not suitable for high-throughput screening (34). The fluorescence-based, quantitative deadenylase assay described here is based on end-point measurement and suitable for 96- and 384-well microplate formats. To show the usefulness of the assay, we screened a small chemical compound library and identified several inhibitors of the Caf1/CNOT7 enzyme. These compounds may be useful tools for the biochemical analysis of the Caf1/CNOT7 deadenylase subunit of the Ccr4–Not complex and indicate the feasibility of developing small molecule inhibitors of Mg^2+^-dependent ribonuclease enzymes as well as the suitability of the fluorescence-based deadenylase assay for the screening of compound libraries.

## MATERIALS AND METHODS

### Plasmids and DNA cloning

A codon-optimized cDNA encoding human Caf1/CNOT7 was generated and subcloned using standard procedures into the bacterial expression plasmid pQE80L (Qiagen) using the BamHI and SalI restriction sites. A codon-optimized cDNA fragment encoding human Ccr4/CNOT6L lacking the amino terminal leucine-rich repeat domain (amino acids 1–155) was obtained using standard polymerase chain reaction techniques and cloned into the multiple cloning site of pQE80L (Qiagen) using the BamHI and SalI restriction endonucleases. The absence of mutations and the appropriate reading frame were confirmed by DNA sequencing. The PARN expression plasmid (pET33PARN) has been described previously (35). Site-directed mutations to inactivate the active sites (D40A, CNOT7, E240A, CNOT6L, D28A and PARN) were introduced using standard protocols (Stratagene Quikchange). Oligonucleotides used for site-directed mutagenesis were designed using the online PrimerX tool (http://www.bioinformatics.org/primerx/).

### Protein expression and purification

The human Caf1/CNOT7 deadenylase enzyme was purified from *Escherichia coli* strain BL21 (DE3). Cells were grown in Lysogeny Broth (LB) medium containing 50 µg/ml ampicillin (2 l) at 37°C with vigorous shaking until the optical density (600 nm) was between 0.6 and 0.8. Expression was then induced by the addition of 0.2 mM isopropyl β-D-1-thiogalactopyranoside for 3 h at 30°C, or overnight at room temperature. Cells were harvested by centrifugation (6000 rpm) using a Sorvall SLC-6000 SUPER-LITE rotor at 4°C for 15 min. The supernatant was discarded and the cell pellet was resuspended in 30 ml ice-cold extraction buffer (20 mM Tris–HCl pH 7.8, 500 mM NaCl, 10% glycerol, 2 mM β-mercaptoethanol). Cells were frozen and kept at −80°C until further use. After thawing the bacterial suspension, the cells were lysed on ice using a Qsonica XL2000 sonicator (amplitude: 40%) using five 30 s on/30 s off cycles. The crude lysate was centrifuged in a Sorvall SS-34 rotor at 10 000 rpm, 4°C for 30 min to remove insoluble material and stored at −80°C until further use.

The hexahistidine-tagged Caf1/CNOT7 protein was purified in a single step using HisTrap columns (GE Life Science; 1 ml bed volume) at 4°C. The soluble lysate was applied to the column using a syringe at an approximate flow rate of 2–3 drops per second (>1 ml/min). Subsequently, the column was washed using a syringe filled with 10 ml wash buffer (20 mM Tris–HCl pH 7.8, 500 mM NaCl, 10% glycerol, 2 mM β-mercaptoethanol, 10 mM imidazole) and finally, eluted with 5 ml elution buffer (20 mM Tris–HCl pH 7.8, 500 mM NaCl, 10% glycerol, 2 mM β-mercaptoethanol, 250 mM imidazole), which was collected in 1 ml fractions. Elution fractions were analysed by sodium dodecyl sulphate–polyacrylamide gel electrophoresis SDS–PAGE and coomassie staining (Invitrogen Bio-Safe Staining kit) and peak fractions used. Ccr4/CNOT6L was expressed and purified from *E. coli* using a similar procedure. The PARN enzyme was purified by immobilized metal affinity chromatography as described before with minor modifications (35).

### Oligonucleotides

Desalted oligonucleotides used as RNA substrate or DNA probe were purchased from Sigma Genosys. Alternatively, high performance liquid chromatography purified oligonucleotides were purchased from Eurogentec. The 16-mer RNA substrate oligonucleotide (5′-CCU UUC CAA AAA AAA A-3′) contained a 5′ fluorescein (Flc) group. The DNA probe (5′-TTT TTT TTT GGA AAG G-3′) contained a 3′ tetramethylrhodamine (TAMRA) or a 3′ black hole quencher (BHQ)-1 modification.

### Assay conditions

Standard reaction conditions for deadenylase assays were: 20 mM Tris–HCl pH 7.9, 50 mM NaCl, 2 mM MgCl_2_, 10% glycerol, 1 mM β-mercaptoethanol and 1.0 µM 5′-Flc-labelled RNA substrate in nuclease-free water.

For gel-based detection of ribonuclease activity, deadenylase reactions (10 µl) were incubated at 30°C for 60 min, stopped by the addition of 12 µl RNA loading buffer [95% formamide, 0.025% bromophenol blue, 0.025% xylene cyanol FF, 0.025% sodium dodecylsulphate and 5 mM ethylenediaminetetraacetic acid (EDTA)] and heated for 3 min at 85°C. A small sample of the RNA mixture (3 µl) was analysed by denaturing PAGE using a 20% acrylamide:bisacrylamide (19:1) gel containing 50% (w/v) urea. Polyacrylamide gels (8 × 8 cm; Invitrogen Xcell system) were pre-run for 30 min at 200 V before sample loading. Flc-labelled RNA was visualized by epifluorescence using a Fujifilm LAS-4000 imager equipped with an Epi-Blue illuminator (460 nm).

For fluorescence-based detection of nuclease activity, deadenylase reactions (10–20 µl) were incubated at 30°C for 60 min and stopped by the addition of an equal volume of probe mix containing 1% SDS, and a 5-fold molar excess of 3′-labelled DNA probe. Fluorescence intensity was measured at 25°C (sensitivity setting 70–90) using a BioTek Synergy HT plate reader with 96 or 384 U-shaped black multi-well plates. Filter sets used were: 485 ± 20 nm (excitation) and 528 ± 20 nm (emission). Data analysis and curve-fitting were carried out using Microsoft Excel 2007 and Graphpad Prism 5.0.

HeLa cytoplasmic extract (S-100 fraction) was purchased from Boston Biochem (cat no F-372, 5 mg/ml).

### Virtual screening

In order to prioritize candidates from our compound collection as likely inhibitors of the Caf1/CNOT7 enzyme, we applied virtual library screening. This collection (University of Nottingham Managed Chemical Compound Collection, MCCC) contains a highly diverse set of 83 086 lead-like compounds. A database containing the chemical structures of the MCCC compounds was prepared for virtual screening using the LigPrep and Epik modules of the Schrödinger (www.schrodinger.com) small-molecule drug discovery software suite (36) in order to standardize protonation states and to generate 3D conformers of the molecules. The coordinates of an X-ray crystal structure of the Caf1–NOT1 complex (25) (Protein Data Bank entry 4GMJ, chain C) were used to construct a docking receptor with Sybyl 8.0 (www.tripos.com) software. Compounds were docked to this receptor using the genetic optimization for ligand docking (GOLD)(37) program (www.ccdc.cam.ac.uk) in standard parameter mode. Docked compound poses were assessed and ranked using Goldscore fitness scores, which ranged from 30 to 88, with high scores (>77) predicting compounds with likely affinity for the active site of the Caf1 enzyme. The top 11% of virtual hits were rescored exhaustively and a total of 1440 predicted hits were selected for bioassay based on fitness scores and plausible binding modes by visual inspection.

### Automated screening

Compounds (10 mM in dimethyl sulphoxide, DMSO) were stored in a dry, inert environment at −20°C and dispensed in 96-well plates using an automated facility (TTP Labtech Technology). Compounds were diluted to 0.5 mM using 20% DMSO/water. Aliquots (4 µl; 25% DMSO/water) were transferred to black U-well 384-well plates (Greiner Bio-One) and subsequent automated screening was carried out using a Biomek 3000 liquid handler (Beckman Coulter). After addition of enzyme (8 µl containing 1.0 µM Caf1/CNOT7, 50 mM Tris–HCl pH 7.9, 125 mM NaCl, 5 mM MgCl_2_, 25% glycerol, 2.5 mM β-mercaptoethanol), plates were left at room temperature for 15 min before the addition of RNA substrate (8 µl). The composition of the final reaction mixture was: 0.4 µM Caf1/CNOT7, 20 mM Tris–HCl pH 7.9, 50 mM NaCl, 2 mM MgCl_2_, 10% glycerol, 1 mM β-mercaptoethanol, 5% DMSO, 100 µM library compound. After incubation at 30°C for 60 min, 20 µl probe mix (5 µM DNA probe, 1% SDS, 20 mM Tris–HCl pH 8.0, 0.5 mM EDTA) was added. Fluorescence was measured using a BioTek Synergy HT plate reader as described above.

## RESULTS

### Design of a fluorescence-based deadenylase assay

**Figure 1.**
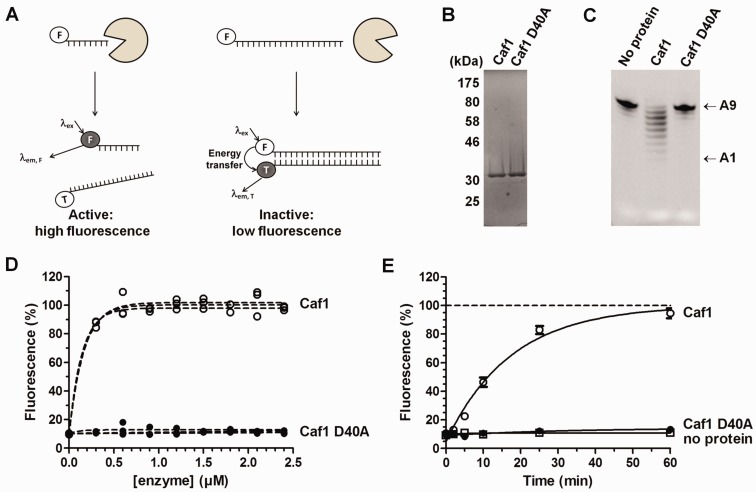
Principle of the fluorescence-based deadenylase assay. (**A**) Schematic diagram of the fluorescence-based deadenylase assay. The assay is based on a 5′ Flc-labelled RNA oligonucleotide substrate. After incubation of the substrate in the presence of a deadenylase enzyme, the reaction is stopped and a 3′ TAMRA-labelled DNA oligonucleotide probe complementary to the RNA substrate is added. Flc fluorescence of intact substrate is quenched upon probe hybridization because of the close proximity of the TAMRA fluorophore. In contrast, the TAMRA-labelled probe cannot hybridize to the Flc-labelled reaction product allowing detection of Flc fluorescence. (**B**) Purified enzymes used for assay development. Wild-type Caf1/CNOT7 and inactive Caf1/CNOT7 containing the amino acid substitution D40A were expressed as *His*-tagged proteins in *E. coli* and purified using immobilized-metal affinity chromatography. Purified proteins (3 µg) were separated by 12% SDS–PAGE and stained with coomassie. (**C**) Gel-based deadenylase assay. Equal amounts of wild-type and inactive Caf1/CNOT7 (0.4 µM) were incubated with 5′ Flc-labelled substrate (1 µM). After incubation (60 min), the substrate RNA was subjected to denaturing PAGE and visualized using epifluorescence. Indicated are the intact RNA substrates containing nine 3′ adenylate residues (A9) and the 8-mer reaction product containing a single 3′ adenylate residue (A1). (**D**) Fluorescence-based measurement of deadenylase activity. The indicated amount of wild-type and inactive (D40A) Caf1/CNOT7 protein was incubated with Flc-labelled substrate (1 µM). After incubation (60 min), a 5-fold molar excess of the 3′ TAMRA-labelled probe was added before fluorescence was measured. (**E**) Measurement of fluorescence as a function of time. Wild-type and inactive D40A Caf1/CNOT7 (0.4 µM) were incubated with Flc-labelled substrate (1 µM). After the indicated time, a 5-fold molar excess of the 3′ TAMRA-labelled probe was added before fluorescence was measured. Error bars indicate the standard error of the mean (*n* = 3).

Based on the findings that short RNA oligonucleotides are substrates of deadenylase enzymes *in vitro* (22,30,38), we designed a substrate detection method based on fluorescence resonance energy transfer (FRET). The two-stage deadenylase assay contained three key components: (i) the (purified) enzyme, (ii) a 5′ fluorophore-labelled RNA substrate and (iii) a DNA probe containing a 3′ fluorophore. We initially used a Flc-labelled RNA substrate and a complementary DNA probe labelled with TAMRA, which form a well characterized ‘FRET pair’ (Figure 1A). The principle of the assay is the complementarity of the probe and the RNA substrate in the absence of deadenylase activity, resulting in close proximity of the Flc and TAMRA fluorophores, which prevents fluorescence of the Flc moiety. In contrast, efficient annealing of the DNA probe is prevented when the substrate is degraded, thus allowing detection of Flc-mediated fluorescence (Figure 1A).

The principle of the assay was tested using purified Caf1/CNOT7, a catalytic subunit of the Ccr4–Not deadenylase complex. Wild-type and catalytically inactive versions of human Caf1/CNOT7 containing an amino-terminal hexahistidine tag were expressed in *E*. *coli* and purified using immobilized-metal affinity purification (Figure 1B). The catalytically inactive version of Caf1/CNOT7 contained the amino acid substitution Asp-40→Ala, which interferes with chelation of Mg^2+^ ions in the active site (22,39,40). Using a gel-based assay, degradation of the substrate was observed when wild-type Caf1/CNOT7 was incubated with a 5′ Flc-labelled 16-mer oligonucleotide substrate containing a 3′ stretch of nine adenosine residues. The deadenylase activity was specific, because it was not observed in the presence of catalytically inactive Caf1/CNOT7 D40A (Figure 1C).

Next, we carried out the fluorescence-based detection of deadenylase activity (Figure 1D). After incubation of the Flc-labelled substrate with Caf1/CNOT7, we added a solution containing sodium dodecylsulphate (0.5% final concentration) to inhibit any residual activity of the Caf1/CNOT7 enzyme (data not shown) and a 5-fold molar excess of the TAMRA-labelled DNA probe. As shown, fluorescence was detected after incubation with wild-type Caf1/CNOT7 and was highly reproducible. In contrast, no fluorescence was observed when the substrate was incubated with inactive Caf1/CNOT7, even in the presence of high enzyme concentrations or when incubated for up to 1 h (Figure 1D and E).

The signal/background ratio was not improved by replacing the TAMRA fluorophore of the probe with a BHQ (Supplementary Figure S1A). To optimize the substrate/probe ratio, we used varying probe concentrations. This indicated that a three-fold probe excess was sufficient to obtain a maximum signal/background ratio (Supplementary Figure S1B).

### Kinetic analysis of deadenylation by Caf1/CNOT7

**Figure 2.**
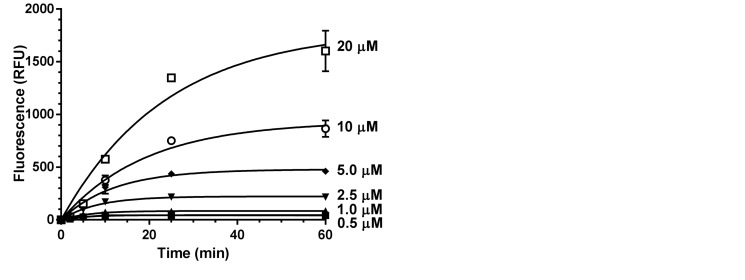
Quantitative analysis of Caf1/CNOT7 enzyme kinetics. (**A**) Measurement of fluorescence as a function of time using the indicated oligonucleotide substrate concentrations. Reactions contained 0.4 µM Caf1/CNOT7 enzyme. Error bars indicate the standard error of the mean (*n* = 3). Fluorescence was normalized by subtraction of background fluorescence observed in the absence of enzyme. (**B**) Kinetic data from panel (A) were plotted to estimate the *K*_m_ by curve fitting of the Michaelis–Menten equation (*K*_m_ = 10.6 ± 2.9 µM). The inset shows the Lineweaver–Burk plot of the kinetic data from panel (A). Curve fitting was carried out using Graphpad Prism. Error bars indicate the standard error of the mean.

To evaluate the suitability of the fluorescence-based deadenylase assay for quantitative analysis, we carried out a kinetic analysis of the Caf1/CNOT7 enzyme activity. Thus, we incubated a fixed amount of Caf1/CNOT7 with increasing substrate concentrations and measured the fluorescence as a function of time (Figure 2A). The results were consistent with multiple substrate turnover events per enzyme. After obtaining the initial rate of reaction by linear regression, the substrate concentration was plotted versus the initial rate of reaction (Figure 2B). By using non-linear regression, we derived the *K*_m_ constant of the Caf1/CNOT7 enzyme for its oligonucleotide substrate (10.6 ± 2.9 µM). Similar values were obtained by linear regression analysis using a Lineweaver–Burke plot (Figure 2B, inset).

### Application of the fluorescence-based deadenylase assay for screening

**Figure 3.**
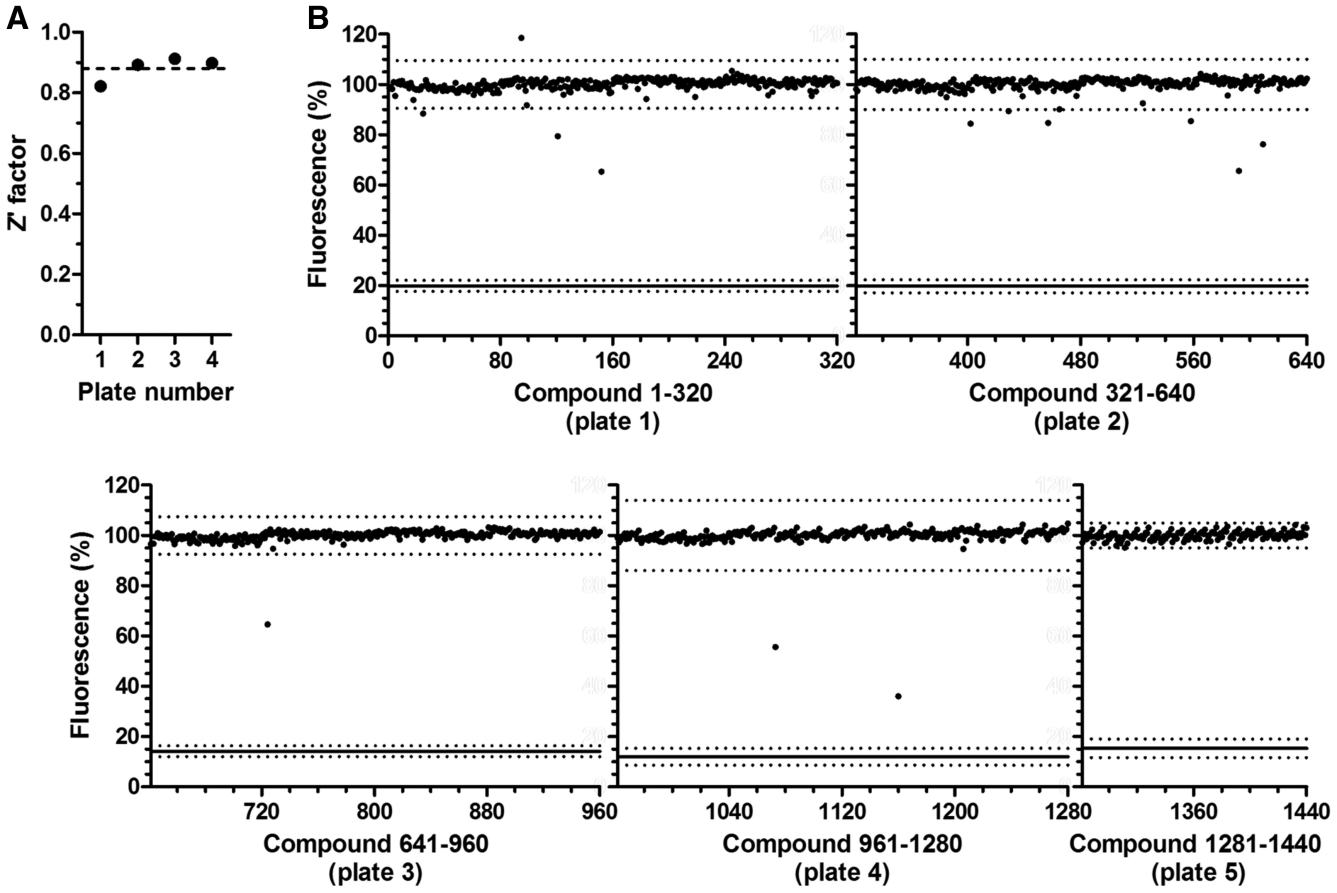
Identification of small molecule inhibitors of Caf1/CNOT7 using a fluorescence-based deadenylase assay. (**A**) Evaluation of the fluorescence-based deadenylase assay for screening by Z factor analysis. The mean value of the Z factor is 0.88 ± 0.02 (*n* = 4). (**B**) Screening of a library of 1440 compounds. The compounds were dispensed in five 384-well plates and pre-incubated with Caf1/CNOT7 enzyme for 15 min at room temperature. After addition of RNA substrate (final concentration: 0.4 µM Caf1/CNOT7 enzyme, 100 µM library compound, 1.0 µM substrate in a reaction volume of 20 µl), reactions were incubated for 60 min. Reactions were stopped by the addition of 20 µl of a solution containing 1.0% SDS and a 5-fold molar excess of probe. Dots indicate fluorescence of each well containing a library compound. Also indicated is the mean background fluorescence (solid line). Dotted lines indicate three standard deviations from the mean of reactions containing library compounds or of the mean background fluorescence, respectively.

To demonstrate the usefulness of the assay, we adapted the fluorescence-based assay for use with 384-well microwell plates and screened a compound library. The screening assay comprises four pipetting steps, which is compatible with automated liquid handling. First, a solution containing a test compound (in 25% DMSO) was dispensed into the microwell plates. Subsequently, a solution containing the Caf1/CNOT7 enzyme was added and incubated for 15 min at room temperature. After addition of the substrate, the reactions were incubated for 60 min at 30°C. Finally, reactions were terminated by the addition of SDS and the DNA probe before measurement of fluorescence. We established that the signal remains stable for up to 7 days when the reactions are kept in the dark at room temperature, which will facilitate the analysis of large numbers of plates in parallel (Supplementary Figure S2). After optimization of automated liquid handling steps, the suitability of the assay for screening was confirmed by determination of the Z factor (0.88 ± 0.02, *n* = 4), which indicates that the assay is of high quality (Figure 3A).

**Figure 4.**
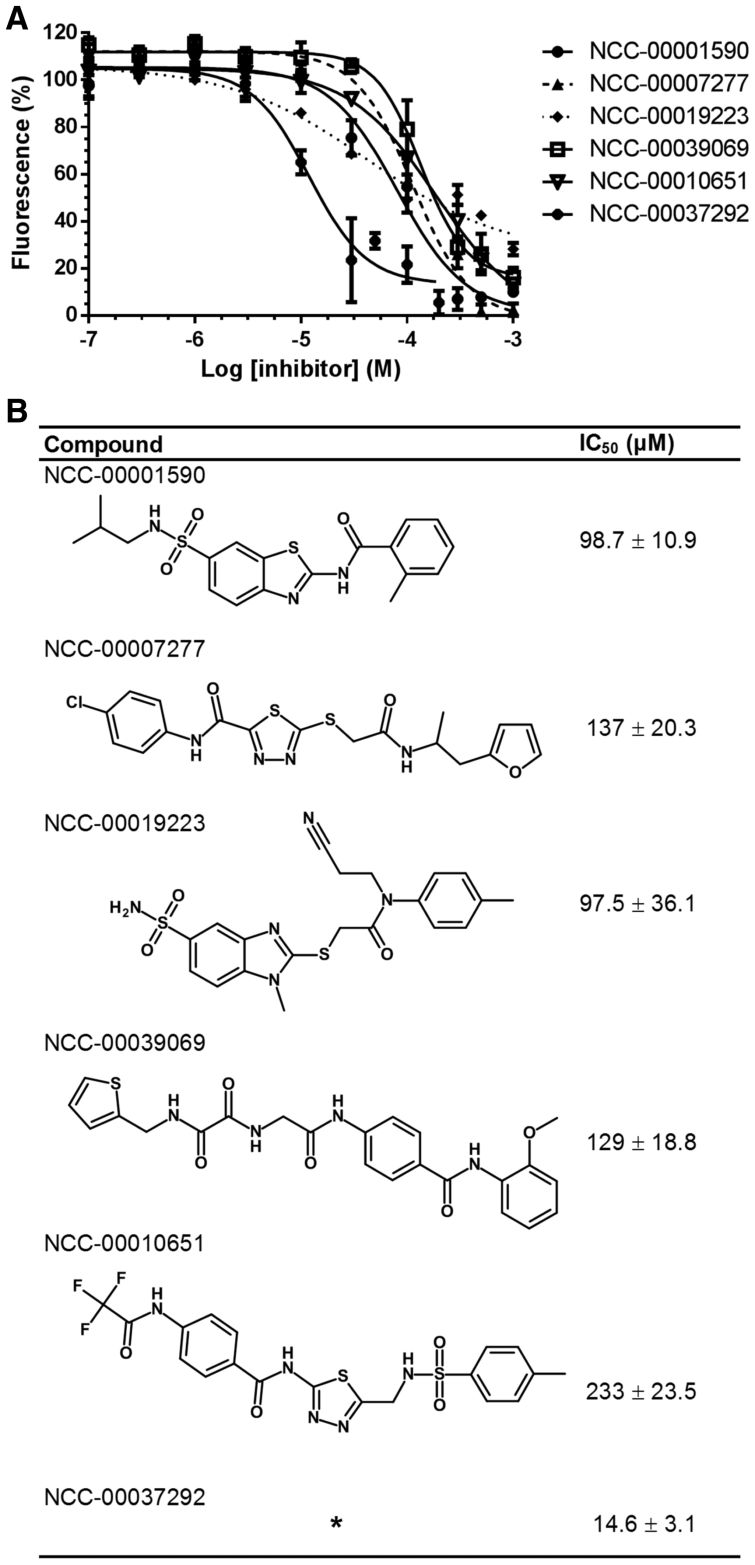
Determination of IC_50_ values of small-molecule inhibitors of Caf1/CNOT7. (**A**) Determination of IC_50_ values. Compounds were pre-incubated with Caf1/CNOT7 for 15 min at room temperature, followed by the addition of RNA substrate. After incubation (60 min at 30°C), reactions were stopped by the addition of SDS and a 5-fold molar excess of probe. Shown are representative experiments. Error bars indicate the standard error of the mean. (**B**) Structures of inhibitors with IC_50_ values <250 µM. The chemical structure of NCC-00037292 will be reported elsewhere. The IC_50_ values shown (± standard error of the mean) are derived from at least three independent replicate experiments.

Subsequently, we assessed the feasibility of identifying small-molecule inhibitors of Caf1/CNOT7 by screening a library of 1440 compounds, which were selected based on a preliminary virtual screening of 83 086 compounds (Figure 3B). This automated procedure led to 11 compounds that were analysed further. Two compounds precipitated and were discarded. Three compounds had very weak inhibitory activity (estimated IC_50_ > 500 µM) and were not investigated further. Of the remaining six, five compounds had IC_50_ values between 100 and 250 µM as determined by using the fluorescence-based deadenylase assay (Figure 4A and B). In addition, we identified one more potent compound with a low micromolar IC_50_ value (Figure 4A and B). The analysis of the structure–activity relationships of a set of analogues of the latter compound is currently underway and will be reported elsewhere.

**Figure 5.**
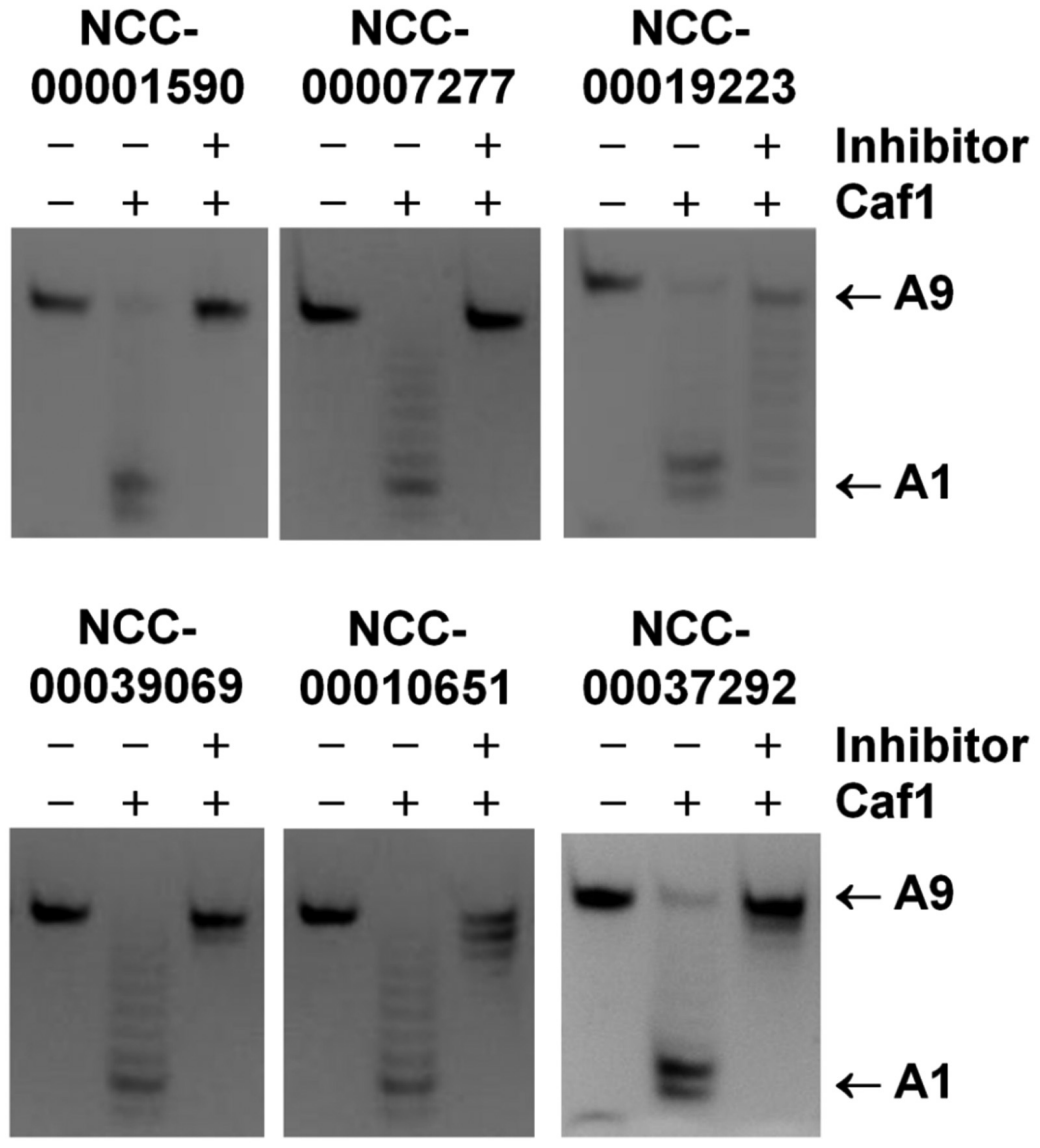
Validation of inhibitory activity using gel-based product analysis. The indicated compounds (NCC-00001590, NCC-00007277, NCC-00019223, NCC-00039069, NCC-00010651, 300 µM; NCC-00037292, 100 µM, final concentration) were incubated with purified Caf1/CNOT7 enzyme (0.4 µM) and the 5′ Flc-labelled oligonucleotide substrate (1.0 µM). After incubation (30°C for 60 min), reactions were inactivated by heating. Products were separated by denaturing PAGE and directly visualized by epifluorescence. Indicated are the intact RNA substrates containing nine 3′ adenylate residues (A9) and the 8-mer reaction product containing a single 3′ adenylate residue (A1).

To ensure that the identified compounds were *bona fide* inhibitors of the Caf1/CNOT7 enzyme, and to exclude the possibility that the compounds were identified based on interference with the fluorescent measurements, we used a gel-based assay. Thus, we incubated the Caf1/CNOT7 enzyme with the oligonucleotide substrate in the presence or absence of the compounds. As expected, Caf1/CNOT7 deadenylated the oligonucleotide substrate, whereas the substrate remained intact in control reactions that did not contain Caf1/CNOT7 enzyme. Importantly, removal of 3′ adenylate residues was greatly reduced in the presence of the identified compounds (Figure 5). Together, these results indicate the suitability of the fluorescence-based deadenylase assay for screening as well as the feasibility of identifying small molecule inhibitors of the Caf1/CNOT7 enzyme.

### Selective inhibition of the Caf1/CNOT7 deadenylase

**Figure 6.**
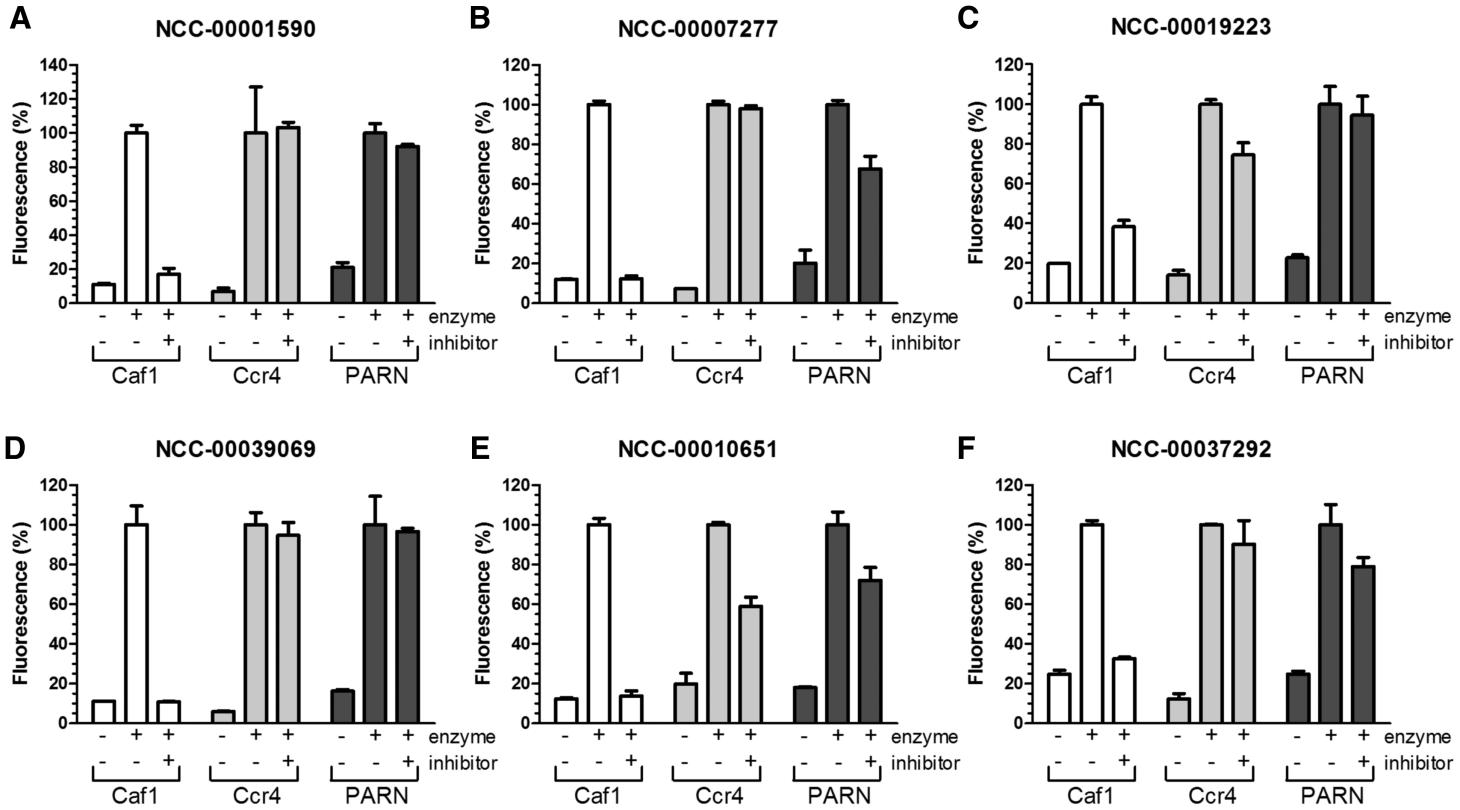
Selective inhibition of the Caf1/CNOT7 deadenylase. The activity of the Caf1/CNOT7, Ccr4/CNOT6L and PARN deadenylase enzymes was assessed in the presence of 300 µM (final concentration) of compound (**A**) NCC-00001590, (**B**) NCC-00007277, (**C**) NCC-00019223, (**D**) NCC-00039069, (**E**) NCC-00010651 and (**F**) NCC-00037292 (100 µM). Enzymes were pre-incubated with the indicated compounds at room temperature for 15 min. After addition of Flc-labelled substrate RNA, reactions were incubated at 30°C for 60 min. Fluorescence was measured after addition of a mixture containing SDS (0.5% final concentration) and a 5-fold molar excess of TAMRA-labelled probe. Error bars indicate the standard error of the mean (*n* = 3).

To assess whether the identified compounds are selective for the Caf1/CNOT7 enzyme, or whether they are more general inhibitors of deadenylase enzymes, we evaluated the effect of the compounds on the activity of the Ccr4/CNOT6L and PARN enzymes. Thus, we incubated these enzymes in the presence or absence of 300 µM of the inhibitors. As shown, compounds NCC-00001590 and NCC-00039069 only inhibited Caf1/CNOT7 and did not inhibit the activity of Ccr4/CNOT6L or PARN (Figure 6A and D). In contrast, compounds NCC-00007277,NCC-00019223 and NCC-00037292 displayed less selectivity and partially inhibited the activity of PARN and Ccr4/CNOT6L, respectively (Figure 6B, C and F). Finally, NCC-00010651 inhibited all three deadenylase enzymes, albeit no complete inhibition of PARN and Ccr4/CNOT6L was obtained (Figure 6E). Taken together, these results indicate that selective inhibition of deadenylase enzymes, specifically of the Caf1/CNOT7 enzyme, with small molecules is feasible. Moreover, the availability of the fluorescence-based deadenylase assay opens up routes for the screening of more extensive compound libraries using automated screening.

### Fluorescence-based detection of 3′ exonuclease activity in complex mixtures

**Figure 7.**
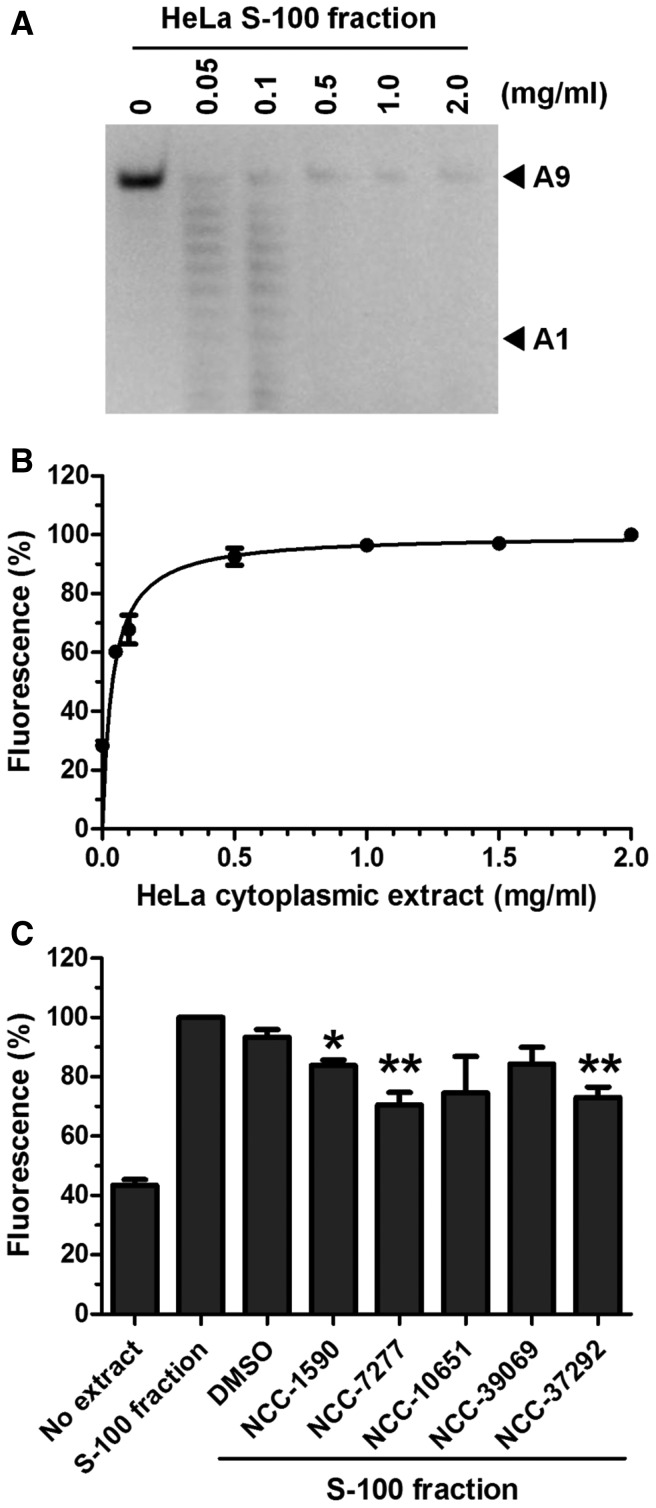
Fluorescence-based detection of 3′ exonuclease activity in complex mixtures. (**A**) Gel-based assay. Increasing amounts of a HeLa S-100 cytoplasmic extract was incubated with 5′ Flc-labelled substrate (1 µM). After incubation (60 min), the substrate RNA was subjected to denaturing PAGE and visualized using epifluorescence. Indicated are the intact RNA substrates containing nine 3′ adenylate residues (A9) and the deadenylated product (A1). (**B**) Fluorescence-based measurement of exonuclease activity. The indicated amount of HeLa S-100 fraction was incubated with Flc-labelled substrate (1 µM). After incubation (60 min), a 5-fold molar excess of the 3′ TAMRA-labelled probe was added before fluorescence was measured. (**C**) The activity of the S-100 fraction was assessed in the presence of NCC-00001590, NCC-00007277, NCC-00019223, NCC-00039069 and NCC-00010651 (300 µM) or NCC-00037292 (100 µM). *P < 0.05, **P < 0.01. Error bars indicate the standard error of the mean (*n* = 3).

To assess the usability of the assay and the identified inhibitors in more complex conditions, we used HeLa S-100 cytoplasmic extracts. First, we incubated increasing concentrations of the S-100 fraction with the Flc-labelled RNA substrate and analysed the reaction products by gel electrophoresis (Figure 7A). At high concentrations (0.5 mg/ml), complete degradation beyond the stretch of nine adenosine residues was observed, as expected, based on the presence of other ribonucleases in the extract. However, a fraction of the RNA substrate appeared resistant against degradation, presumably because of RNA-binding proteins. When we used the fluorescence-based detection, we observed a clear dose–response effect with >90% of maximal signal observed in the presence of 0.5 mg/ml S-100 fraction (Figure 7B). To determine the activity of the identified inhibitors of Caf1 in the context of a complex mixture, we used a subsaturating amount of S-100 fraction (0.05 mg/ml). Comparison of the signal obtained in the absence or presence of S-100 fraction indicated that a high background was observed when using a crude extract. This is consistent with the observation that a fraction of the RNA substrate remains refractory to degradation in these conditions. Although addition of DMSO (5%) did not significantly inhibit activity of the extract, partial inhibition was observed in the presence of three compounds. The strongest effect was observed with compounds NCC-00007277 and NCC-00037292 (Figure 7C). Interestingly, these compounds partially inhibit PARN (Figure 6), which is the predominant deadenylase in cell extracts (41,42).

## DISCUSSION

Here we report a new method for the analysis of the biochemical activity of deadenylase enzymes. The fluorescence-based deadenylase assay is sensitive, can be used for quantitative analysis and is suitable for miniaturization using 96- and 384-well plates. We believe that this assay has significant advantages for the quantitative evaluation of deadenylase enzymes over existing approaches, such as analyses based on gel electrophoresis, methylene blue colourimetry or size-exclusion chromatography (32–34). Due to the sensitivity of the fluorescence-based deadenylase assay, activity can be detected at much lower concentrations and in smaller reaction volumes as compared with colourimetry- or chromatography-based assays. Moreover, the assay is fast and much less laborious as compared with methods involving gel electrophoresis or chromatography. Combined with its suitability for plate-based formats, this allows the evaluation of a large number of reactions in parallel with less effort as compared with any of the alternative methods.

We demonstrate the use of the fluorescence-based deadenylase assay for the screening of compound libraries and identified one compound with relatively high affinity (IC_50_ between 10 and 20 µM) and five inhibitors with relatively low potency (IC_50_ around 100 µM). Despite their low potency, these compounds will be useful for the biochemical analysis of deadenylase enzymes. The identified inhibitors are structurally unrelated, but, based on preliminary molecular modelling analysis, we believe that all compounds bind in the active site thereby blocking interactions with the RNA substrate. A process to derive a structure–activity relationship is currently underway for the most potent compound NCC-00037292. In addition, the screening of more extensive compound collections in combination with the synthesis and evaluation of novel chemical entities will likely result in more potent inhibitors of the Caf1/CNOT7 deadenylase enzyme that can be appraised in cell-based assays. This will also provide more detail about binding to the Caf1 deadenylase and the mechanism of inhibition.

Recently, Balatsos and co-workers (43,44) reported inhibitors of the PARN deadenylase, which—as is the case with Caf1/CNOT7—contains a DEDD domain. In contrast to the compounds reported here, the reported inhibitors of the PARN enzyme were nucleoside analogues with *K*_i_ values ranging between 20 and >500 µM. It will be of interest to establish whether these nucleoside analogues are selective inhibitors of PARN or whether they also inhibit other deadenylases such as Caf1/CNOT7.

In conclusion, we believe that the fluorescence-based deadenylase assay described here complements existing assays for the quantitative, biochemical analysis of deadenylase enzymes, e.g. when comparing the activities of wild-type enzymes with those containing amino acid substitutions. By using the assay for the screening of a compound library, we demonstrate the utility of the assay as well as the feasibility of developing selective inhibitors of the Caf1/CNOT7 deadenylase subunit of the Ccr4–Not complex. Such inhibitors, together with inhibitors of other Mg^2+^-dependent ribonucleases, such as those inhibiting PARN (43,44), will be highly useful tools as chemical probes that complement existing resources available for the study of post-transcriptional gene regulation.

## SUPPLEMENTARY DATA

Supplementary Data are available at NAR Online.

## FUNDING

The Medical Research Council [G1100205]; Islamic Development Bank [merit scholarship programme to M.M.]. Funding for open access charge: University of Nottingham; Research Councils UK (MRC).

*Conflict of interest statement*. None declared.

## Supplementary Material

Supplementary Data
