# Supplementary material for: A fluorescence-based assay suitable for quantitative analysis of deadenylase enzyme activity
Source: Nucleic Acids Res. 2013 Oct 28;42(5):e30. doi: 10.1093/nar/gkt972 (PMC3950723; doi:10.1093/nar/gkt972)
Supplement: Supplementary Data [file supp_42_5_e30__index.html]

A fluorescence-based assay suitable for quantitative analysis of deadenylase enzyme activity — A fluorescence-based assay suitable for quantitative analysis of deadenylase enzyme activity — Supplementary Data 

# A fluorescence-based assay suitable for quantitative analysis of deadenylase enzyme activity

## Supplementary Data

files

**Files in this Data Supplement:**

- Supplementary Data - pdf file
