# Supplementary material for: A fluorescence-based assay suitable for quantitative analysis of deadenylase enzyme activity
Source: Nucleic Acids Res. 2013 Oct 28;42(5):e30. doi: 10.1093/nar/gkt972 (PMC3950723; doi:10.1093/nar/gkt972)
Supplement: Supplementary Data [file supp_gkt972_nar-01795-met-g-2013-File009.pdf]

Supplementary Data

A fluorescence-based assay suitable for quantitative analysis of deadenylase enzyme activity

Maryati Maryati, Ishwinder Kaur, Jadhav Gopal, Blessing Oveh, Lubna Hashmi, Peter M Fischer and G Sebastiaan Winkler

School of Pharmacy and Centre for Biomolecular Sciences, University of Nottingham, University Park, Nottingham NG7 2RD, United Kingdom

Figure S1

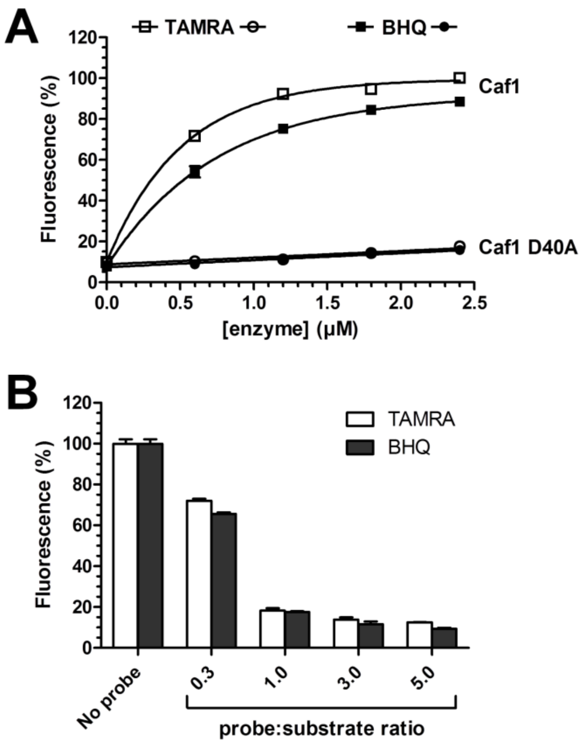

Figure S1. Probe optimisation.
(A) Comparison of probes containing a 3′ TAMRA or a black hole quencher (BHQ)-1 moiety. The Flc-labelled RNA substrate (1.0 µM) was incubated for 60 min at 30 ºC in the presence of the indicated amount of Caf1/CNOT7 enzyme. Reactions were stopped by the addition of SDS (final concentration of 0.5%) and a five-fold molar excess of the indicated probe. (B) Optimisation of probe:substrate ratio. The 5′ Flc-labelled RNA substrate (1.0 µM) was incubated for 60 min at 30 ºC in the presence of Caf1/CNOT7 enzyme (0.4 µM). Reactions were stopped by the addition of SDS (final concentration of 0.5%) and the indicated molar excess of a probe containing either a TAMRA or BHQ-1 modification of the 3′ end of the oligonucleotide. Error bars indicate the standard error of the mean.

Figure S2

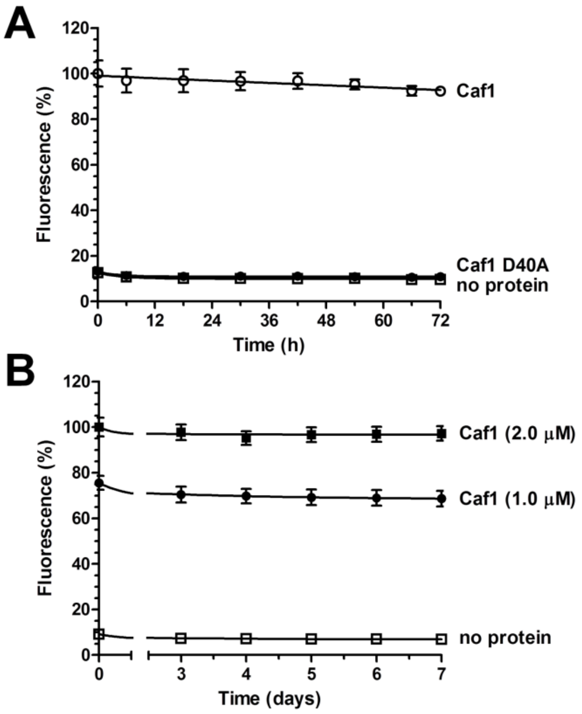

Figure S2. Signal stability.
(A) Stability of fluorescence intensity measured up to 72 h after the completion of reactions. Reactions containing Flc-labelled substrate were incubated for 60 min at 30 ºC before the addition of a solution containing SDS (final concentration of 0.5%) and a five-fold excess of 3′ TAMRA labelled probe. Fluorescence was measured at regular intervals 0-72 h after addition of the probe mixture. Reactions were kept at room temperature in the dark. (B) The fluorescence signal remains stable up to 7 days after addition of the probe mixture. Reactions containing Flc-labelled substrate were incubated for 60 min at 30 ºC before the addition of a solution containing SDS (final concentration of 0.5%) and a five-fold excess of 3′ TAMRA labelled probe. Fluorescence was measured immediately after addition of the probe mix and at 24 h intervals from day 3 until day 7. Reactions were kept at constant temperature (20 ºC) in the dark. Error bars indicate the standard error of the mean.
